# Validation of reference genes as an internal control for studying *Avena sativa–Puccinia coronata* interaction by RT-qPCR

**DOI:** 10.1038/s41598-022-18746-z

**Published:** 2022-08-26

**Authors:** Sylwia Sowa, Magdalena Sozoniuk, Joanna Toporowska, Krzysztof Kowalczyk, Edyta Paczos-Grzęda

**Affiliations:** Institute of Plant Genetics, Breeding and Biotechnology, University of Life Sciences in Lublin, Akademicka 13, 20-950 Lublin, Poland

**Keywords:** Genetics, Molecular biology

## Abstract

In this study we evaluated eleven candidate reference genes in *Avena sativa* during compatible and incompatible interactions with two different pathotypes of *Puccinia coronata* f. sp. *avenae* in six time points post-inoculation. The identification of genes with high expression stability was performed by four algorithms (geNorm, NormFinder, BestKeeper and ΔCt method). The results obtained confirmed that the combination of two genes would be sufficient for reliable normalization of the expression data. In general, the most stable in the tested plant-pathogen system were *HNR* (heterogeneous nuclear ribonucleoprotein 27C) and *EF1A* (elongation factor 1-alpha). *ARF* (ADP-ribosylation factor) and *EIF4A* (eukaryotic initiation factor 4A-3) could also be considered as exhibiting high expression stability. *CYP* (cyclophilin) was shown by all assessment methods to be the worst candidate for normalization in this dataset. To date, this is the first report of reference genes selection in *A. sativa–P. coronata* interaction system. Identified reference genes enable reliable and comprehensive RT-qPCR analysis of oat gene expression in response to crown rust infection. Understanding the molecular mechanisms involved in the host–pathogen interactions may expand knowledge of durable resistance strategies beneficial to modern oat breeding.

## Introduction

*Avena sativa* L. is a species cultivated on a global scale with a high range of applications from the food industry to pharmaceuticals and animal feed^1,2^. One of the greatest threats to cultivated oat are fungal diseases, of which the most common is crown rust caused by *Puccicnia coronata* f. sp *avenae* P. Syd. & Syd^3,4^. Research on the *Puccinia* population biology confirms the high virulence dynamics of this pathogen^5,6^. Despite the identification of over 100 crown rust resistance genes (*Pc*), due to the high adaptive potential of the fungus, most of them have already been overcome. Hence, the need to conduct the research on obtaining durable plant resistance, effective under conditions favourable to the disease development^7,8^. Genetically determined disease resistance mechanisms, although universal in nature, are very complex and closely dependent on the plant-pathogen system. Plant immune studies are often based on the analysis of gene expression profiling with the use of modern molecular biology methods, of which qPCR (quantitative PCR, real-time PCR) has become very popular due to its high sensitivity and specificity. In order to obtain highly reliable and repeatable results, the MIQE (minimum information for publication of quantitative real-time PCR experiments) guideline was developed recommending the standardization of each step of the qPCR experiment^9^. In accordance with this laboratory practice, reliable qPCR experiment requires the selection of at least two reference genes with a stable level of expression in a given experimental system. The housekeeping genes of basic cellular metabolism are widely used for this purpose, however numerous studies have shown that none of these genes are universal^10^. The stability of the reference gene expression may vary depending on the species studied, tissue type, developmental stage or experimental conditions^11–14^. For this reason, it is crucial to analyze the expression stability of potential reference genes individually for each experimental system. This type of research was carried out mainly under abiotic stress conditions. Jaiswal et al.^15^ analyzed the expression of ten candidate reference genes during seed development in *Cyamopsis tetragonoloba* (L.) Taub. Similar studies were conducted in *Rhizophora apiculata*^16^, *Hordeum brevisubulatum*^17^ *Hypericum perforatum*^18^ and *Hordeum vulgare*^19^. In the genus *Avena*, studies involving the selection of reference genes to analyse the gene expression under various types of stress have so far been conducted mainly on *Avena fatua* L. Wrzesińska et al.^20^ assessed the expression of six genes (18S ribosomal RNA (*18S rRNA*), actin (*ACT*), TATA-binding protein (*TBP*), α-tubulin (*TUB*), elongation factor 1-alpha *(EF1A*), and glyceraldehyde-3-phosphate dehydrogenase (*GAPDH*) in *A. fatua* biotypes resistant to various types of herbicides. Liu et al.^21^ attempted to evaluate eight candidate reference genes (*18S rRNA*, *28S rRNA*, *ACT*, *GAPDH*, *EF1A*, ribosomal protein L7 (*RPL7*), α-*TUB*, and *TBP*) in *A. fatua* differend types of tissues at various growth stages under the influence of the herbicide. Similar studies verifying the four reference genes (*ACT*, *EF1A*, *GAPDH*, *TBP*) in herbicide treated stem and leaf tissues were carried out on *A. ludoviciana*^22^. The stability of reference genes was also tested for the molecular analysis of the *A. fatua* caryopses dormancy^23^. However, the large size (12.5 Gb) of the allohexaploid genome (AACCDD) and the basic chromosome number of 2n = 6x = 42 make *A. sativa* a very difficult research object^24^. Oat genome structure may indicate the presence of mainly duplicated genes. This hinders the finding of the proper RGs or design of optimal primers, as each copy of the duplicated gene may not be uniformly expressed in different samples^25,26^. Preferably “single-copy” genes should be used as a reference, however, the study of Yang et al.^27^ provides a proof of concept that using duplicated RG is also feasible and valid in polyploid oat.

The study attempts to determine the reference genes for the *A. sativa–P. coronata* experimental setup. The obtained results will provide valuable data necessary for a comprehensive analysis of gene expression in oat. The study will also constitute a reference point for further analyses of the new *Pc* genes identified in our previous research^28–31^.

## Materials and methods

### Plant material and crown rust inoculation

The study material consisted of oat line Pc39 (Pendek × Pc39) with major gene resistance to crown rust caused by *P. coronata* f. sp. *avnae* and the Polish cultivar Kasztan (Dawid × CHD 1685/84) susceptible to rust infection^32^. The differential near-isogenic line Pc39 was developed at the Cereal Research Centre AAFC Winnipeg, Canada^33–35^. The line carries a major race-specific crown rust resistance gene *Pc39* incorporated into common oat from the wild hexaploid oat *Avena sterilis* F-366 collected in Israel^36^. Seeds of the studied genotypes were grown in plug trays filled with a universal substrate containing peat for 10 days in a phytotron at 18 °C with a 16-h photoperiod.

Two *P. coronata* f. sp. *avenae* pathotypes used in the study (13.1, 230, Table 1) with a virulence profile defined based on the standard differentials set^37^ and supplemental differentials (*Pc14*, *Pc35*, *Pc36*, *Pc55*, *Pc57*, *Pc60*, *Pc61*, *Pc63*, *Pc67*, *Pc70*, *Pc71*, *Pc91*, *Pc94*, *Pc96*, *Pc97*, *Pc98*, *Pc101*, *Pc103-1* and *Pc104*) were selected from a collection of single-pustule isolates derived from populations collected in Poland in the years 2010–2019^6,38,39^. Both pathotypes are virulent to ‘Kasztan’. Pc39 line is resistant to 13.1 and susceptible to 230. Dried pathotypes were stored in 1.5-ml microfuge tubes at − 70 °C. Before inoculation, urediniospores were heat-shocked for 4 min at 42 °C and multiplied on leaf fragments of the susceptible oat cultivar Kasztan^32^ using the host–pathogen method of Hsam et al.^40^, originally used for *Blumeria graminis* f. sp. *avenae* and modified by Paczos-Grzęda and Sowa^6^. Study was conducted on the first leaves of 10-day-old seedlings. Inoculation was performed in a settling towers by spreading uredinospores on plant material at a density of ≈ 200 spores/cm^2^. Pc39 line was inoculated using both *P. coronata* pathotypes and ‘Kasztan’ was inoculated with 230 *P. coronata* pathotype. Glass slides were placed between the inoculated leaves to monitor the inoculation density. The settling towers were thoroughly cleaned with ethanol between inoculations with different isolates and the room sprayed with water. Seedlings were incubated in a spore-proof growth chamber at 18 °C with 70% humidity, light intensity of approximately 4 kLx under a 16-h photoperiod.

**Table 1:** Virulence spectrum of *P. coronata* f. sp. *avenae* pathotypes used for inoculation.

Race no	Phenotype code^a^	Virulence to supplemental differentials
13.1	BLBG	Pc55, Pc98, Pc103-1
230	LQBC	Pc35, Pc36, Pc60, Pc61, Pc63, Pc70, Pc91

^a^Phenotype code based on the standard differentials set.

Plant experiments were performed in accordance with relevant guidelines and regulations.

### RNA extraction and reverse transcription

RNA extraction was performed after 0 (uninoculated ‘Kasztan’ and Pc39), 8, 16, 24, 48 and 72 h post inoculation (hpi). Three biological replicates, each composed of five leaves of different seedling from the study group were sampled. After harvesting, plant material was immediately frozen in liquid nitrogen and grind to a fine powder with sterile mortar and pestle. Total RNA was extracted with TRIzol reagent (Invitrogen) according to the manufacturer’s instructions. The integrity and quality of RNA samples were evaluated by electrophoresis on 1.5% agarose gel stained with ethidium bromide. The RNA quantity was determined with NanoDrop2000 spectrophotometer (Thermo Fisher Scientific Inc., USA). Prior to cDNA synthesis, potential genomic DNA contamination was eliminated by DNase I (EURx Ltd., Poland) treatment of all RNA samples at 37 °C for 30 min and heat inactivated at 65 °C for 10 min with 50 mM EDTA. The cDNA synthesis was performed in 20 µl reaction volume using the NG dART RT kit (EURx Ltd., Poland), 2 µg RNA sample and oligo (dT)18 primer, as per manufacturer’s instructions. cDNA samples were diluted with nuclease-free water and used for RT-qPCR.

### Selection of reference genes and primer design

Eleven candidate reference genes frequently used in other plants: *ARF* (ADP-ribosylation factor), *CYP* (cyclophilin), *EF1A* (elongation factor 1-alpha), *EIF4A* (eukaryotic initiation factor 4A-3), *GAPDH* (glyceraldehyde-3-phosphate dehydrogenase), *HNR* (heterogeneous nuclear ribonucleoprotein 27C), *HSP70* (heat shock protein 70), *TUA* (alpha tubulin), *UBC* (ubiquitin conjugating enzyme), *ACT* (actin), *EP* (expressed protein) were chosen for expression stability assessment^41,42^. Specific primers for qPCR (Table S1) were obtained based on existing literature^20,27,43^ as well as designed with PrimerBLAST tool (http://www.ncbi.nlm.nih.gov/tools/primer-blast/). Homologous oat sequences were retrieved from the *A. sativa* v2.0 genome detabase (https://wheat.pw.usda.gov/jb?data=/ggds/oat-ot3098v2-pepsico) and oat seed transcriptome^44^ via BLASTn search using highly conserved regionsof the corresponding *Triticum aestivum*, *Avena fatua*, and *Brachypodium distachyon* cDNA sequences with an expected value (E) < 10^−10^ and minimum base identity > 95% as blast criteria with CLC Genomics Workbench (CLCbio, Seoul, Korea).

### Quantitative realtime PCR analysis of candidate reference genes

For qRT-PCR, primer specificity was determined using melting curve analysis and the PCR products were evaluated on 2% agarose gel. The amplification efficiency (E) and regression coefficients (R^2^) of candidate reference genes were calculated based on the standard curve generated by a fivefold serial dilution points of cDNA combined with a mix containing all the studied samples. The calculation of E values is as follows: E (%) = (10^−1/slope^ − 1) × 100. Only specific primer pairs with efficiency ranging from 90 to 110%, the slope between − 3.6 to − 3.1 and correlation coefficient (R^2^) over 0.99 were left for further analysis.

Diluted aliquots of the reverse-transcribed cDNAs were used as templates in qPCR assays. Quantitative PCR was performed in three biological replicates with three technical replicates, no template control (NTC) and no reverse transcription control (NRT) on QuantStudio™ 3 Real-Time PCR System (Applied Biosystems, USA) with Power Track SYBR Green Master Mix (Thermo Fisher Scientific Inc., USA). Each 20 μL reaction mixture contained 20 ng of cDNA 1 × qPCR Mix and 400 nM of each primer. The qPCR program was as follows: 95 °C for 2 m, 40 cycles of 95 °C for 15 s, 60 °C for 1 m. To confirm the amplification specificity and lack of primer dimer formation, each run was performed with a melting curve analysis.

### Analysis and validation of gene expression stability

The raw data of qPCR was processed by means of Thermo Fisher Scientific online data analysis app. The expression stability of the selected housekeeping genes was analysed using either untransformed Cq values for BestKeeper^45^ and ΔCt method or relative quantities for NormFinder^46^ and geNorm^47^ software packages. The input data included reaction efficiency corrections. The expression stability analysis was conducted on five datasets—separately for *A. sativa*–*P. coronata* compatible and incompatible interactions, independently for Kasztan cultivar and Pc39 oat line and additionally as full dataset consisting of all experimental samples^48^.

For validation of identified RGs the relative expression of the target gene encoding Phenylalanine ammonia lyase (*PAL*) (Table S1) was analysed. qPCR reaction was conducted as described earlier. Data normalization was performed with two most stable RGs identified for given dataset (individually and in combination) and with the least stable gene. The relative mRNA level was calculated according to the 2^−∆∆Ct^ method^48^ with uninoculated samples being used as calibrator.

## Results

### Primer specificity and efficiency check

**Figure 1:**
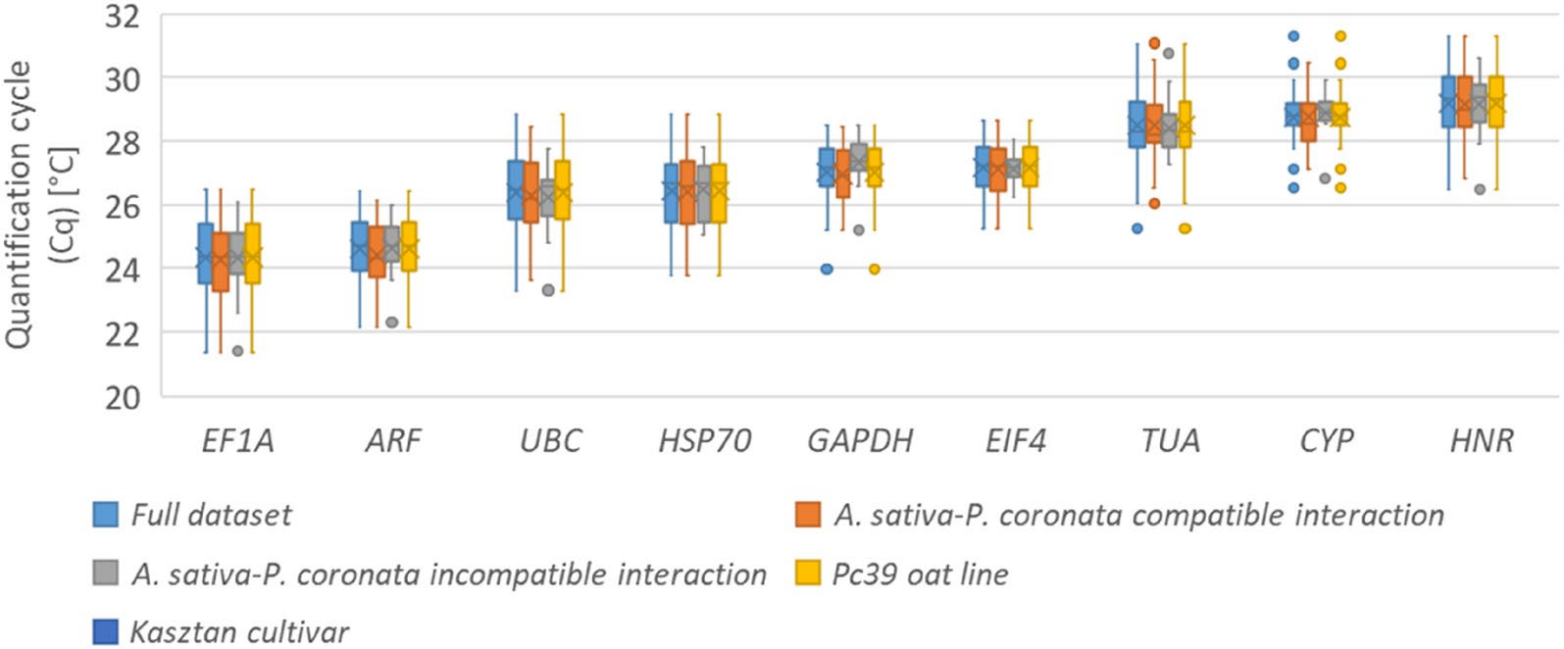
Cq values for nine candidate reference genes *(ARF* (ADP-ribosylation factor),*CYP (*cyclophilin), *EF1A* (elongation factor 1-alpha), *EIF4A* (eukaryotic initiation factor 4A-3), *GAPDH* (glyceraldehyde-3-phosphate dehydrogenase), *HNR* (heterogeneous nuclear ribonucleoprotein 27C), *HSP70* (heat shock protein), *TUA* (alpha tubulin), and *UBC* (ubiquitin conjugating enzyme (E2)) across experimental samples. A line across the box is depicted as the median. The box indicates the 25th and 75th percentiles, the dots represent outlier values.

To identify the most stable reference genes combination, cDNA of tested oat genotypes was used in the qPCR. The primers specificity was estimated by qPCR melting curve analysis and further evaluated on 2% agarose gel. Single peaks were generated on the dissociation curves obtained for each primer pair thus confirming the specificity of the amplification. Moreover, single desired size bands were observed on agarose gel (Fig. S1–S2). No signal was detected in the no template control (NTC) and no reverse transcription control (NRT). PCR amplification efficiency ranged from 90.26% (*EIF4A*) to 128.7% (*EP)* and the R^2^ based on linear regression varied from 0.875 to 0.997. Primer pairs for *ACT* an *EP* did not meet the adopted effectiveness criteria (90% < E < 110%; R^2^ > 0.99), so they were excluded from the analysis (Table 2). The Tm values of the remining primer pairs varied from 83.8 °C (*EF1A*) to 89.5 °C (*CYP*), and the amplicon sizes were between 88(*EIF4A*) and 170 bp (*EF1A*) (Table S1). The raw quantification cycle (Cq) values were estimated for determination of the gene expression levels. The Cq values for analysed samples ranged between 21.37 and 32.29 (Fig. 1).

**Table 2:** Parameters derived from RT-qPCR analysis—slope, regression coefficient (R^2^), reaction efficiency and melting temperature of the amplicon (Tm).

Gene	Slope	R^2^	Efficiency (%)	Tm (°C)
*ARF*	− 3.34	0.993	99.32	85.6
*CYP*	− 3.21	0.994	104.93	89.5
*EF1A*	− 3.58	0.992	90.26	83.8
*EIF4A*	− 3.16	0.991	107.11	86.4
*GAPDH*	− 3.28	0.997	101.62	85.9
*HNR*	− 3.41	0.994	96.42	83.9
*HSP70*	− 3.12	0.997	109.19	86.6
*TUA*	− 3.42	0.996	96.11	83.9
*UBC*	− 3.09	0.997	110.22	84.4
*ACT*	− 2.93	0.987	119.47	84.1
*EP*	− 2.78	0.875	128.69	79.6
*PAL*	− 3.21	0.996	104.79	86.6

### geNorm analysis

**Figure 2:**
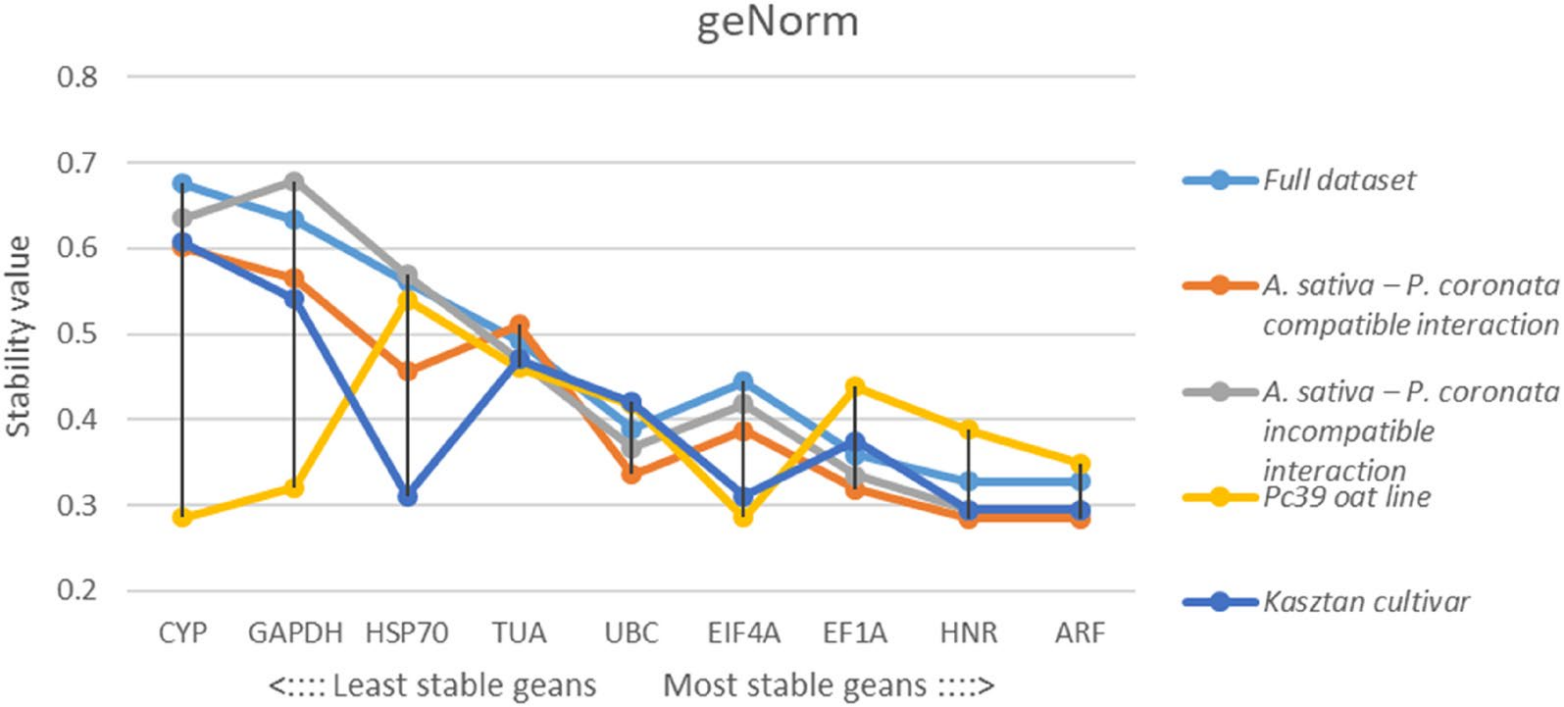
Expression stability values (M) of tested RGs determined by geNorm algorithm for full dataset, compatible interaction dataset, incompatible interaction dataset, Pc39 oat line dataset and Kasztan cultivar dataset. Low M values indicate high expression stability across samples set.

Candidate RGs were ranked by geNorm algorithm according to the stability value M. The cut-off threshold established by Vandesompele et al.^47^ for M value was set at 1.5, with lowest M values being exhibited by genes showing the highest expression stability. All tested genes in all analyzed datasets showed high expression stability (M < 0.7). For all samples analyzed in *A. sativa*–*P. coronata* interaction system *ARF* and *HNR* displayed the most stable expression, while *CYP* showed the least stable expression. Identical results were obtained for compatible interaction dataset. Incompatible interaction subgroup yielded contrary results with *CYP* being one of the most stable among the RGs tested. The best-performing RGs in Kasztan cultivar were *EIF4A* and *HSP70*, whereas most stable RGs found in Pc39 oat line were correspondingly *ARF* and *HNR* (Fig. 2).

### NormFinder analysis

**Figure 3:**
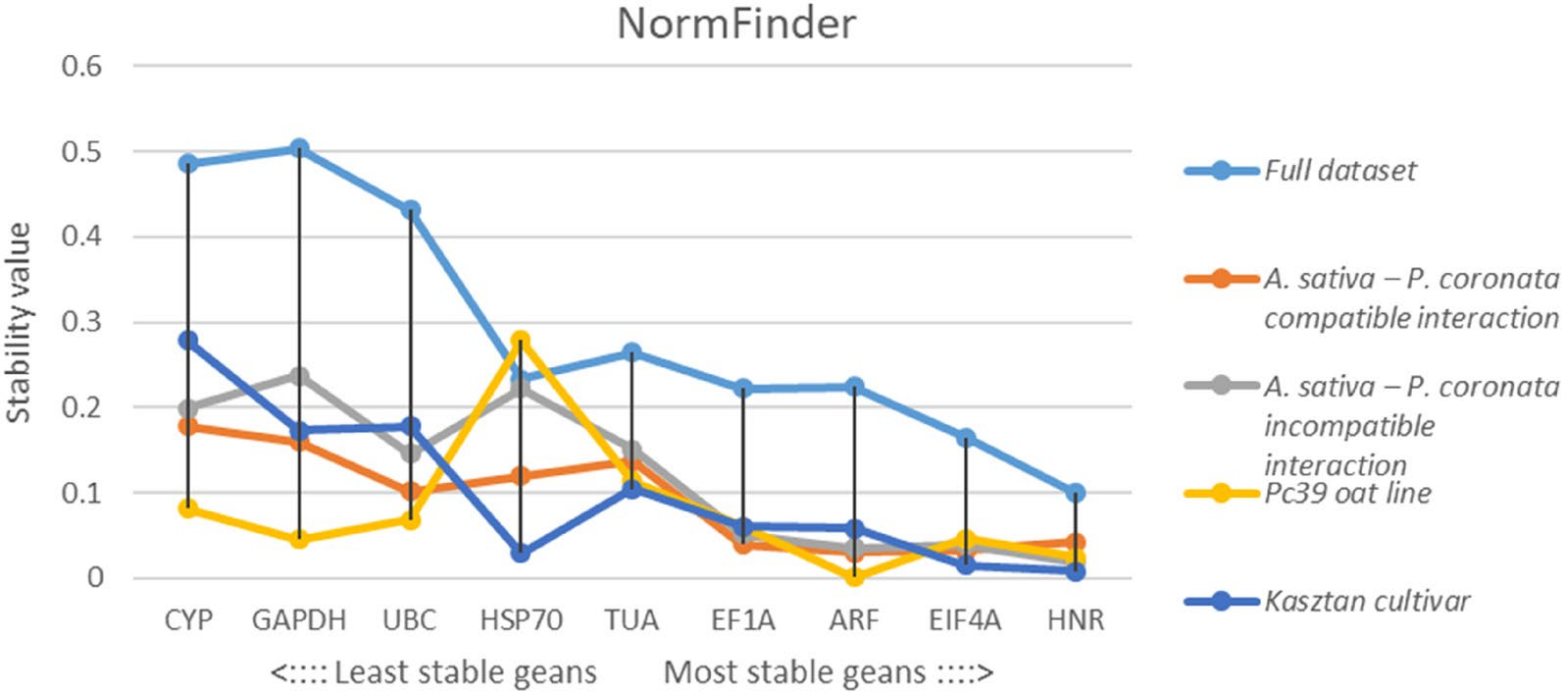
Stability values (SV) of tested RGs determined by NormFinder algorithm for full dataset, compatible interaction dataset, incompatible interaction dataset, Pc39 oat line dataset and Kasztan cultivar dataset. Low SV values indicate high expression stability across samples set.

Stability value (SV) of each candidate RGs was estimated using NormFinder algorithm. RGs with low SV are considered best candidates for qPCR data normalization. Overall, *HNR* and *EIF4A* were two most stable RGs in analyzed pathosystem. When compatible and incompatible interactions were evaluated separately, *ARF* and *EIF4A* or *ARF* and *HNR* were best-scoring RGs pairs, respectively. The highest SVs representing high variation in expression were reported for *GAPDH* and *CYP* in the total dataset as well as in compatible interaction subgroup. Within incompatible interaction subgroup maximum variation was displayed by *HSP70* and *TUA* (Fig. 3).

### BestKeeper analysis

**Figure 4:**
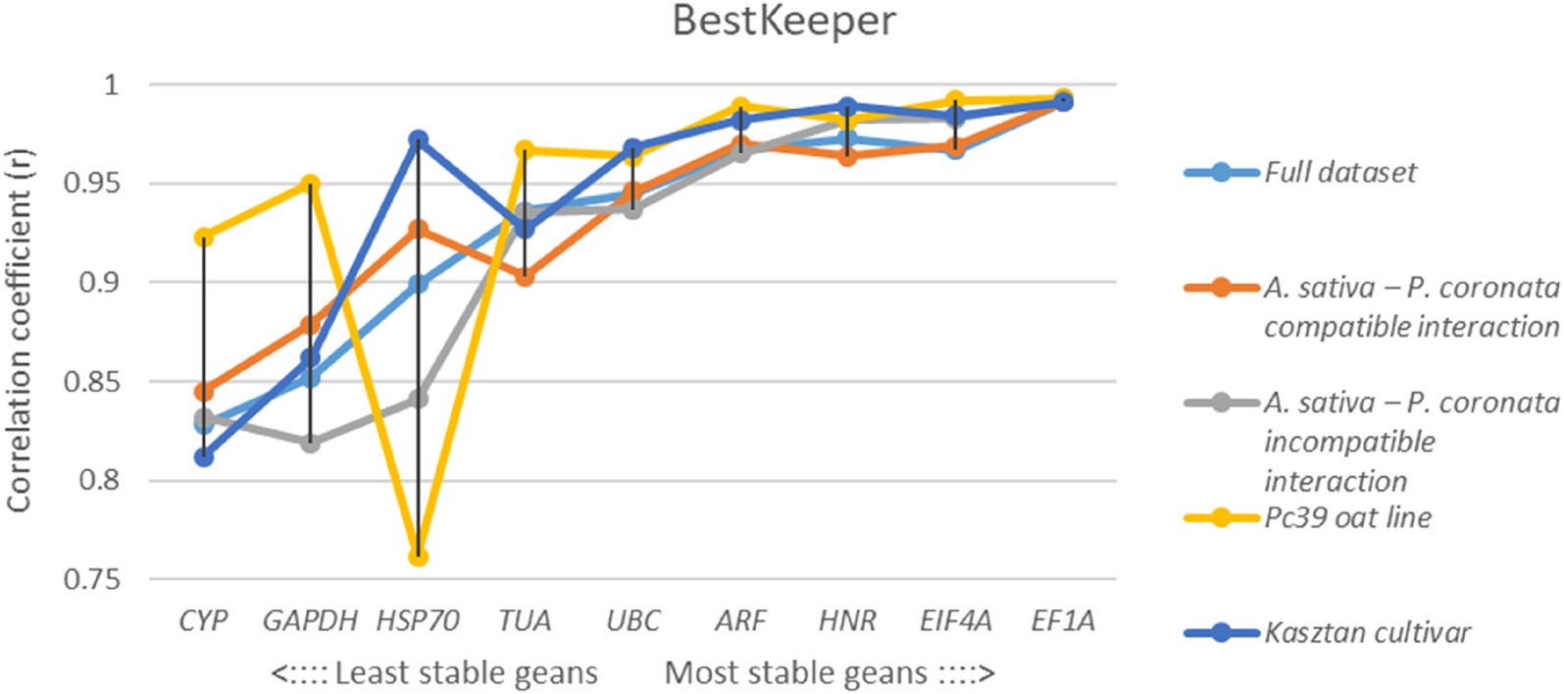
Correlation coefficients (*r*) of tested RGs determined by BestKeeper algorithm for full dataset, compatible interaction dataset, incompatible interaction dataset, Pc39 oat line dataset and Kasztan cultivar dataset. High coefficient of correlation indicates high expression stability across samples set.

According to BestKeeper’s correlation coefficients (*r*) the most stably expressed gene in all experimental datasets (regardless of the samples subgroups being analyzed separately or together) was *EF1A*. High correlations were also obtained for *HNR* (total dataset and Kasztan samples set), *EIF4A* (incompatible subgroup and Pc39 samples set) and *ARF* (compatible subgroup). Out of all genes tested *CYP* was found to be the worst or second-worst performing RG (Fig. 4) in each analyzed samples set.

### ΔCt method

**Figure 5:**
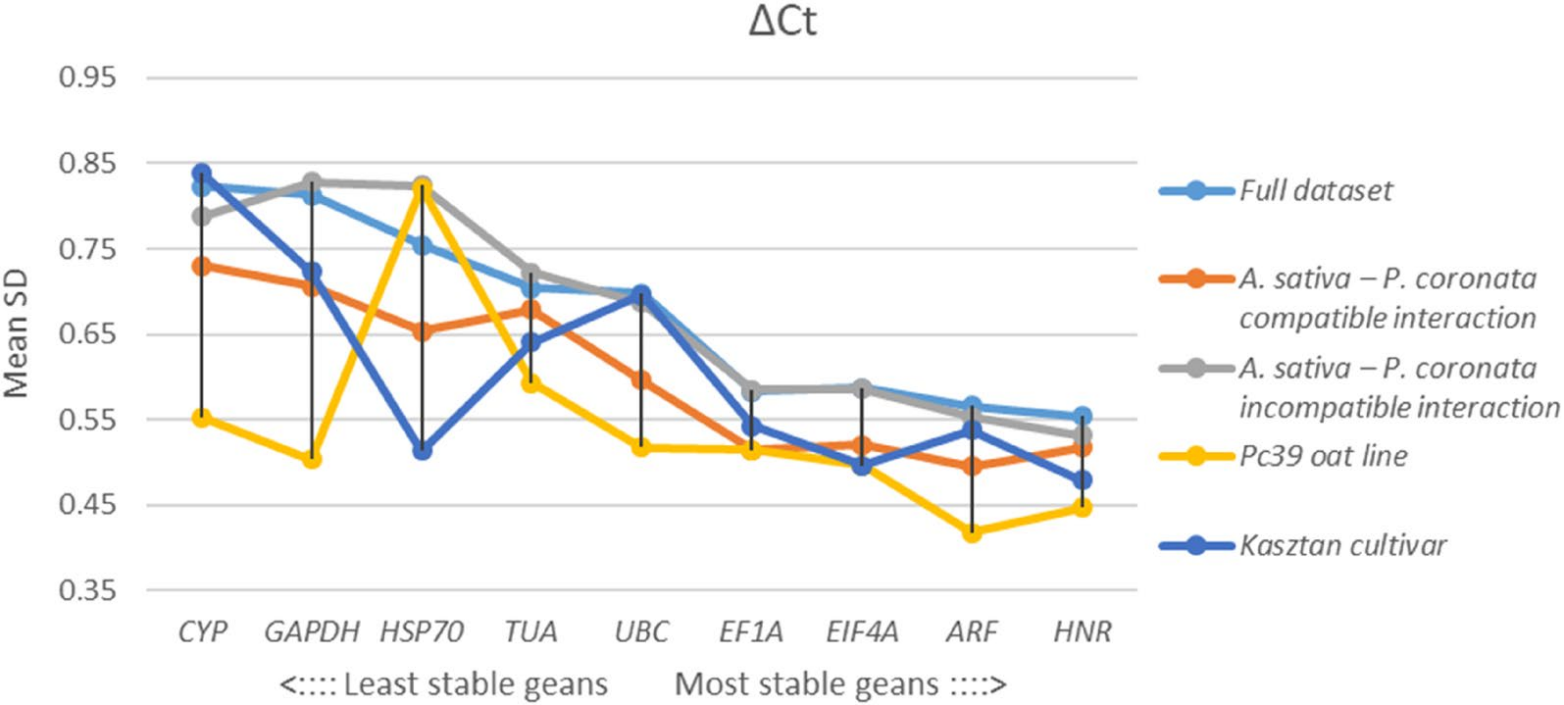
Mean standard deviation (mean SD) of tested RGs determined by ΔCt method for full dataset, compatible reaction dataset, incompatible reaction dataset, Pc39 oat line dataset and Kasztan cultivar dataset. Low average SD indicate high expression stability across samples set.

Based on average standard deviation (mean SD) generated by ΔCt method *HNR* and *ARF* were identified as the most stable RGs for the entire dataset. Similar results were obtained for incompatible interaction samples as well as for Pc39 oat line samples. For compatible interaction subgroup *ARF* and *EF1A* were found to be the least variable RGs, whereas across Kasztan cultivar samples the lowest mean SD was reported for *HNR* and *EIF4A* (Fig. 5).

### Determination of the optimal number of RGs for data normalization

**Figure 6:**
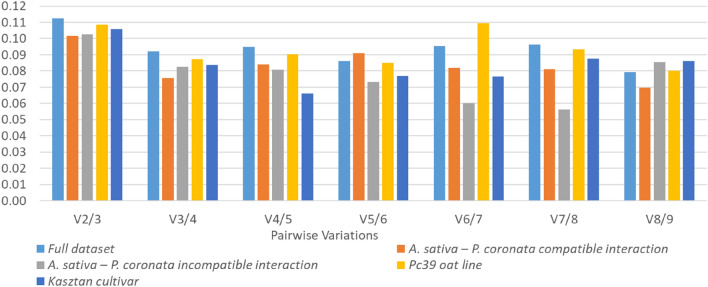
Pairwise variation (Vn/Vn + 1) analysis performed by geNorm algorithm for determination of the optimal number of RGs for accurate qPCR data normalization. A value < 0.15 indicates no need for additional RG inclusion.

Pairwise variation (V_n_/V_n+1_) calculated by geNorm algorithm allows the prediction of optimal number of RGs that should be used for accurate qPCR data normalization. The V_n_/V_n+1_ value below 0.15 indicates that the inclusion of additional RG will not significantly improve reliability of data analysis. In terms of pairwise variation the V_2_/_3_ values calculated within all analyzed datasets were below 0.12 (Fig. 6). Consequently, regardless of the samples being analyzed together or in the various subgroups, only two best-performing RGs are required for sufficient data normalization.

Candidate RGs rankings generated by all tested algorithms are shown in Table 3. In the present study *HNR* and *EF1A* could be considered as most stable in tested plant-pathogen system. Their ranking positions, however, slightly differ depending on calculation method. High level of expression stability was also displayed by *ARF*. All algorithms conclusively pointed out to *GAPDH* and *CYP* as the least stable pair of RGs in combined samples dataset.

**Table 3:** Ranking of candidate RGs stability according to tested algorithms for whole *A. sativa*–*P. coronata* pathosystem and specific samples subgroups.

Method	< –- Most stable genes Least stable genes –- >								
	1	2	3	4	5	6	7	8	9
All samples combined									
geNorm	*ARF/HNR*	–	*EF1A*	*UBC*	*EIF4A*	*TUA*	*HSP70*	*GAPDH*	*CYP*
NormFinder	*HNR*	*EIF4A*	*EF1A*	*ARF*	*HSP70*	*TUA*	*UBC*	*CYP*	*GAPDH*
BestKeeper	*EF1A*	*HNR*	*EIF4A*	*ARF*	*UBC*	*TUA*	*HSP70*	*GAPDH*	*CYP*
ΔCt method	*HNR*	*ARF*	*EF1A*	*EIF4A*	*UBC*	*TUA*	*HSP70*	*GAPDH*	*CYP*
*A. sativa–P. coronata* compatible interaction									
geNorm	*ARF/HNR*	–	*EF1A*	*UBC*	*EIF4A*	*HSP70*	*TUA*	*GAPDH*	*CYP*
NormFinder	*ARF*	*EIF4A*	*EF1A*	*HNR*	*UBC*	*HSP70*	*TUA*	*GAPDH*	*CYP*
BestKeeper	*EF1A*	*ARF*	*EIF4A*	*HNR*	*UBC*	*HSP70*	*TUA*	*GAPDH*	*CYP*
ΔCt method	*ARF*	*EF1A*	*HNR*	*EIF4A*	*UBC*	*HSP70*	*TUA*	*GAPDH*	*CYP*
*A. sativa–P. coronata* incompatible interaction									
geNorm	*CYP/EIF4A*	–	*GAPDH*	*ARF*	*HNR*	*UBC*	*EF1A*	*TUA*	*HSP70*
NormFinder	*ARF*	*HNR*	*GAPDH*	*EIF4A*	*EF1A*	*UBC*	*CYP*	*TUA*	*HSP70*
BestKeeper	*EF1A*	*EIF4A*	*ARF*	*HNR*	*TUA*	*UBC*	*GAPDH*	*CYP*	*HSP70*
ΔCt method	*ARF*	*HNR*	*EIF4A*	*GAPDH*	*EF1A*	*UBC*	*CYP*	*TUA*	*HSP70*
Pc39 oat line									
geNorm	*ARF/HNR*	–	*EF1A*	*UBC*	*EIF4A*	*TUA*	*HSP70*	*CYP*	*GAPDH*
NormFinder	*HNR*	*ARF*	*EIF4A*	*EF1A*	*UBC*	*TUA*	*CYP*	*HSP70*	*GAPDH*
BestKeeper	*EF1A*	*EIF4A*	*HNR*	*ARF*	*UBC*	*TUA*	*HSP70*	*CYP*	*GAPDH*
ΔCt method	*HNR*	*ARF*	*EF1A*	*EIF4A*	*UBC*	*TUA*	*CYP*	*HSP70*	*GAPDH*
Kasztan cultivar									
geNorm	*EIF4A/HSP70*	–	*HNR*	*ARF*	*EF1A*	*UBC*	*TUA*	*GAPDH*	*CYP*
NormFinder	*HNR*	*EIF4A*	*HSP70*	*ARF*	*EF1A*	*TUA*	*GAPDH*	*UBC*	*CYP*
BestKeeper	*EF1A*	*HNR*	*EIF4A*	*ARF*	*HSP70*	*UBC*	*TUA*	*GAPDH*	*CYP*
ΔCt method	*HNR*	*EIF4A*	*HSP70*	*ARF*	*EF1A*	*TUA*	*UBC*	*GAPDH*	*CYP*

In general, *ARF* and *EF1A* were among top best ranked RGs in compatible interaction subgroup, whereas in incompatible interaction subgroup it was *ARF* in combination with *EIF4A*. The latter dataset produced most incongruent rankings with some RGs (such as *EF1A* or *CYP*) being ranked by various algorithms as either showing best, medium or even poor expression stability. Irrespective of this, *HSP70* was shown by all assessment methods to be the worst candidate for normalization in this dataset.

Both *HNR* and *ARF* were indicated as most stable in Pc39 oat line samples. Nonetheless, in Kasztan cultivar samples *HNR* and *EIF4A* were found to be best candidates for internal controls. In this material, as opposed to other datasets, *HSP70* was shown to exhibit relatively high expression stability.

### Expression analysis of target gene for reference genes validation

Aiming to validate the reliability of the selected RGs, the expression analysis of *PAL* gene was performed in experimental samples harvested in various time points post inoculation. For each dataset, both best and worst performing RGs were used in order to demonstrate how incorrect data normalization may affect obtained results.

**Figure 7:**
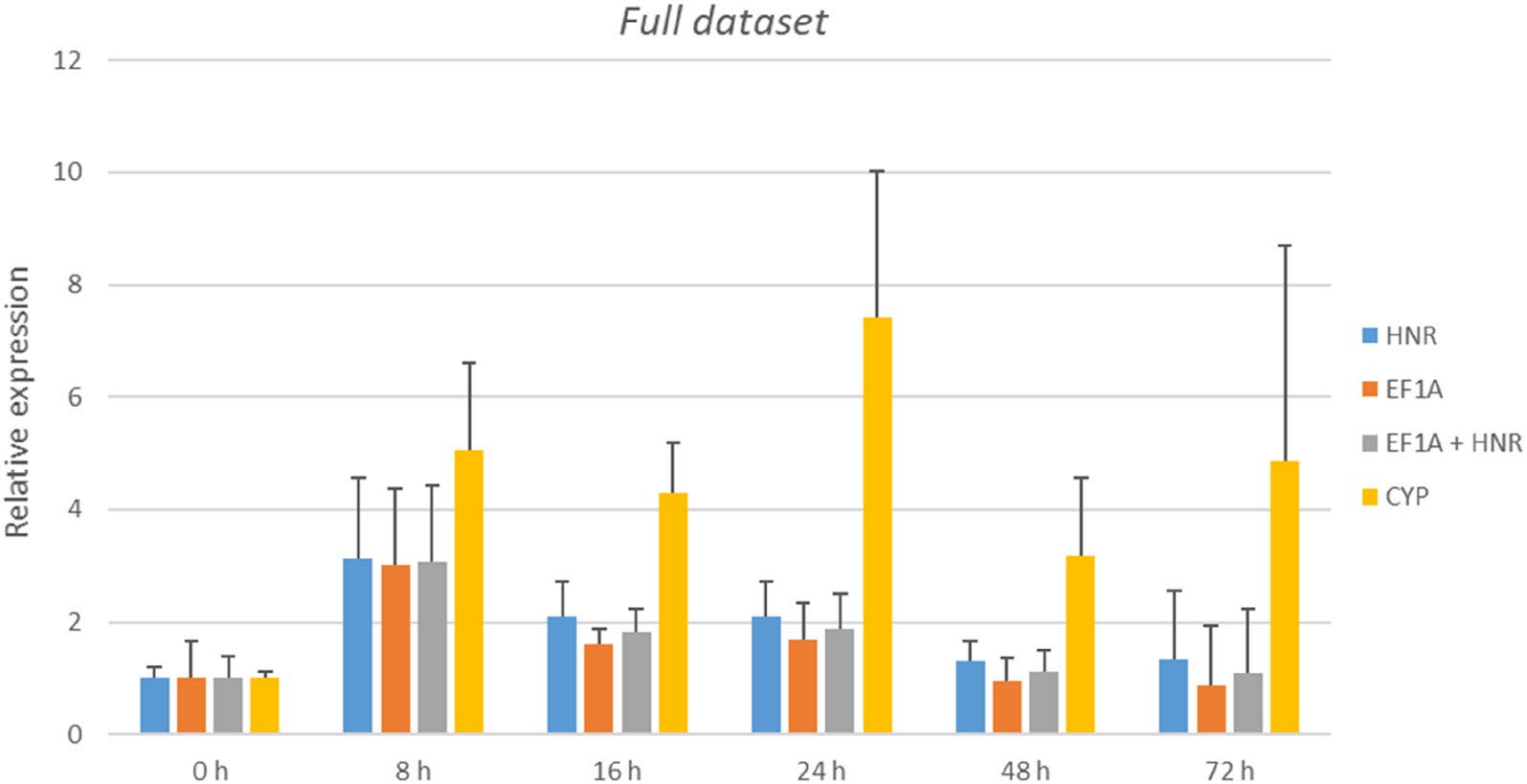
Relative expression of the *PAL* gene following *P. coronata* f. sp.*avenae* inoclulation. Analysis was performed for full dataset normalized against two best performing RGs (*HNR* and *EF1A*) separately or together as well as against worst performing RG (*CYP*). Data is shown as mean ± SD.

When normalization was carried out with most stable RGs (used either individually or in pair) consistent expression patterns were observed in every time point in all datasets (Fig. 7). However, when RGs showing poor stability were used, a clear overestimation of transcript level was noticed. This was especially true for samples collected 24 hpi. Results from full dataset normalized against best RGs (either *HNR*, *EF1A* or *HNR* + *EF1A*) show approximately twofold increase in transcript abundance. These results are distorted when *CYP* is used as RG, suggesting strong transcription upregulation (sevenfold increase). In consequence it shows how inaccurate and altered obtained expression profiles might be if RGs selection is not carried out properly.

### Discussion

Studying complex plant resistance mechanisms is based on RNA sequencing (RNAseq) and transcriptome analysis using high-throughput next-generation sequencing approaches (NGS). Accurate quantification and validation of gene expression derived from in silico NGS data is performed by reverse transcription quantitative real-time polymerase chain reaction. This research aimed to identify reference genes with stable expression level in order to ensure obtaining repeatable and reliable RT-qPCR data.

Eleven frequently used candidate reference genes were evaluated in *A. sativa* during interactions with two different pathotypes of *P. coronata* in six time points post inoculation. The use of crown rust races with defined virulence profiles allowed to stimulate susceptibility as well as resistance response and observe compatible and incompatible interactions respectively. Previous research involving the selection of reference genes in the genus *Avena* enabled the use of specific primers for qPCR developed in these studies^20,27^. The design of the remaining primers was facilitated by the use of recently released *A. sativa* v2.0 genome (“*Avena sativa*—OT3098 v2, PepsiCo,” n.d.) and available, although significantly limited *Avena* transcriptomic data^44^.

In this study nine of eleven candidate reference genes were subjected to further analysis. Primer pairs for actin (*ACT*) and expressed protein (*EP*) were excluded for not reaching the efficiency criterion of 90 to 110%. Actin is one of the typically used housekeeping reference genes, however many studies testing larger panels of potential RGs eliminate this gene due to its low expression stability^49^. Yet, in the genus *Avena ACT* was chosen as suitable for qPCR data normalisationin *A. fatua*and *A. ludoviciana* under herbicide treatment^21,22^. Nevertheless, under the biotic stress of *Trichoderma polysporum* infection in *A. fatua*, *ACT* did not perform very well^50^ in contrast to*TBP* (TATA-binding protein), *18S rRNA* (18S ribosomal RNA) and *UBC*, that were the most stable internal reference genes in this pathosystem. Moreover, according to Wrzesińska et al.^20^ in the study of herbicide-resistant *A. fatua* biotypes *TBP* and *GAPDH* were chosen as the most stably expressed reference genes instead of *ACT*.

Remaining RGs expression stability was assessed with four algorithms, geNorm, NormFinder, BestKeeper and ΔCt method. For each algorithm, RGs were ranked from the most to least stable. Nonetheless, due to the different calculation methods, our data indicated a slight variation depending on the used algorithm.

GeNorm and NormFinder require relative quantities of Cq values (RQ). GeNorm calculates a stability measure (M-value) by the stepwise exclusion of the least stable gene and assesses the pairwise variation (V_n_/V_n+1_) between two sequential normalization factors that contain increasing numbers of genes^47^. The use of this algorithm allowed to determine the optimal number of reference gene combinations. Because the pairwise variation of V_2_/_3_ values for all of the experimental sets was lower than the cut-off threshold of 0.15, this number, in the case of the *A. sativa–P. coronata* pathosystem may be limited to two best-performing RGs. Similar results were obtained by Ruduś et al.^23^ in the study examining the best reference genes for molecular studies of dormancy in wild oat (*A. fatua* L.) caryopses. Based on the pairwise variation analysis, further confirmed by the validation experiment, two RGs may be sufficient for reliable normalization of the expression data. Moreover, the authors concluded that the use of two or three reference genes of high expression stability, performed better than a single gene, which is also in line with the MIQE guideline^9^.

In NormFinder analysis, intra- and intergroup variation within subgroups of a full dataset is calculated with ANOVA-based model. In this study experimental design allowed to divide the dataset into four subgroups: compatible reaction, incompatible reaction, Pc39 oat line and Kasztan cultivar dataset. However, in order to use ANOVA model, assumptions concerning homogeneity of variance and normality of data must be made^51^. In this analysis at the subgroup level, SV of all tested RGs was below the default limit of 0.5 indicating relatively high expression stability^52^. In the total dataset, the highest variation in expression was reported for *GAPDH*, *CYP* and *UBC*, however the value of SV above 0.5 was reported only for *GAPDH*. *GAPDH* was commonly used as an endogenous control in expression analysis in response to biotic^53^ and abiotic stress^54,55^ while many studies of potential RGs evaluation confirm its expression variability^49^. In the *Triticum aestivum*–*Puccinia triticina* pathosystem analyzed by Prasad et al.^56^,*GAPDH* was also the least stable of the five tested housekeeping genes along with *18S rRNA*. The study was conducted on the wheat leaves during pre-haustorial stages of plant-pathogen compatible and incompatible interaction.

In contrast, for BestKeeper and ΔCt method, Cq values with efficiency corrections were used. BestKeeper calculates the geometric mean of Cq values and the most stable genes are indicated by high correlation coefficients and low standard deviations^45^. Simple ΔCt is based on comparisons between each RG and the other RGs within each sample and calculates the average standard deviation against the other RGs^57^. In this study, both methods indicate identical order for the five genes with the lowest expression stability (*CYP*, *GAPDH*, *HSP70*, *TUA*, *UBC*), whereas differences appear when a comparison of the most stable genes is made. BestKeeper ranks *EF1A* as one with the highest correlation coefficient, however the large standard deviation of the results for this gene should also be taken into account. The results of the ΔCt method place *EF1A* fourth out of nine in the stability ranking. According to this method, *HNR* and *ARF* were identified as the most stable RGs for the entire dataset.

In general, the most stable in the tested plant-pathogen system were *HNR* (heterogeneous nuclear ribonucleoprotein 27C) and *EF1A* (elongation factor 1-alpha). *ARF* (ADP-ribosylation factor) and *EIF4A* (eukaryotic initiation factor 4A-3) could also be considered as exhibiting high expression stability. Similar results were obtained by Yang et al.^27^, who evaluated the expression of eleven genes in *A. sativa* developing seeds and corresponding endosperm, as well as shoots and roots of seedlings. They have chosen *EIF4A* + *HNR* as the best performing candidate RG set across all tested samples and in developing endosperms. *EF1A* was among the best RGs for developing seeds. They have also pointed out *GAPDH* as the least stable RG which confirms this study results.

In this study, the worst candidate for normalization regardless of the assessment method was *CYP* (cyclophilin). *CYP* was tested as a candidate reference gene in the study of Tajti et al.^43^ validating RGs for studying different abiotic stresses in oat by RT-qPCR and was ranked as one of the least stable genes under drought stress, however had high stability in leaves under salt stress. *CYP* expression was also analyzed in the research of Wang et al.^58^ on *Nitraria tangutorum* seedlings under a series of experimental conditions and was ranked as one with the average stability. The least suitable as reference gene in the abovementioned experiment was *HSP70*, which in our study also had very low expression stability. This indicates the possible involvement of *HSP70* in plant biotic interaction^59^.

The valdation of RGs carried out in this research through *PAL* expression analysis confirmed the need of using carefully chosen RGs for data normalization. The use of stable internal controls produced consistent expression patterns. It was also shown that resorting to unstable RGs could result in data misinterpretation.

To our best knowledge, this is the first report regarding reference genes selection in *A. sativa*–*P. coronata* interaction system. The obtained results will provide valuable data necessary for a comprehensive analysis of oat gene expression in response to crown rust infection. This may significantly contribute to the understanding of the complex plant resistance mechanisms involved in the host–pathogen interactions and expand knowledge of durable resistance strategies beneficial to modern oat breeding.

## Supplementary Information

Supplementary Information.

## Data Availability

The datasets generated during and/or analyzed during the current study are available from the corresponding author on reasonable request.
