# Supplementary material for: Validation of reference genes as an internal control for studying Avena sativa–Puccinia coronata interaction by RT-qPCR
Source: Sci Rep. 2022 Aug 26;12:14601. doi: 10.1038/s41598-022-18746-z (PMC9418433; doi:10.1038/s41598-022-18746-z)
Supplement: Supplementary file 1 — Supplementary Information. [file 41598_2022_18746_MOESM1_ESM.pdf]

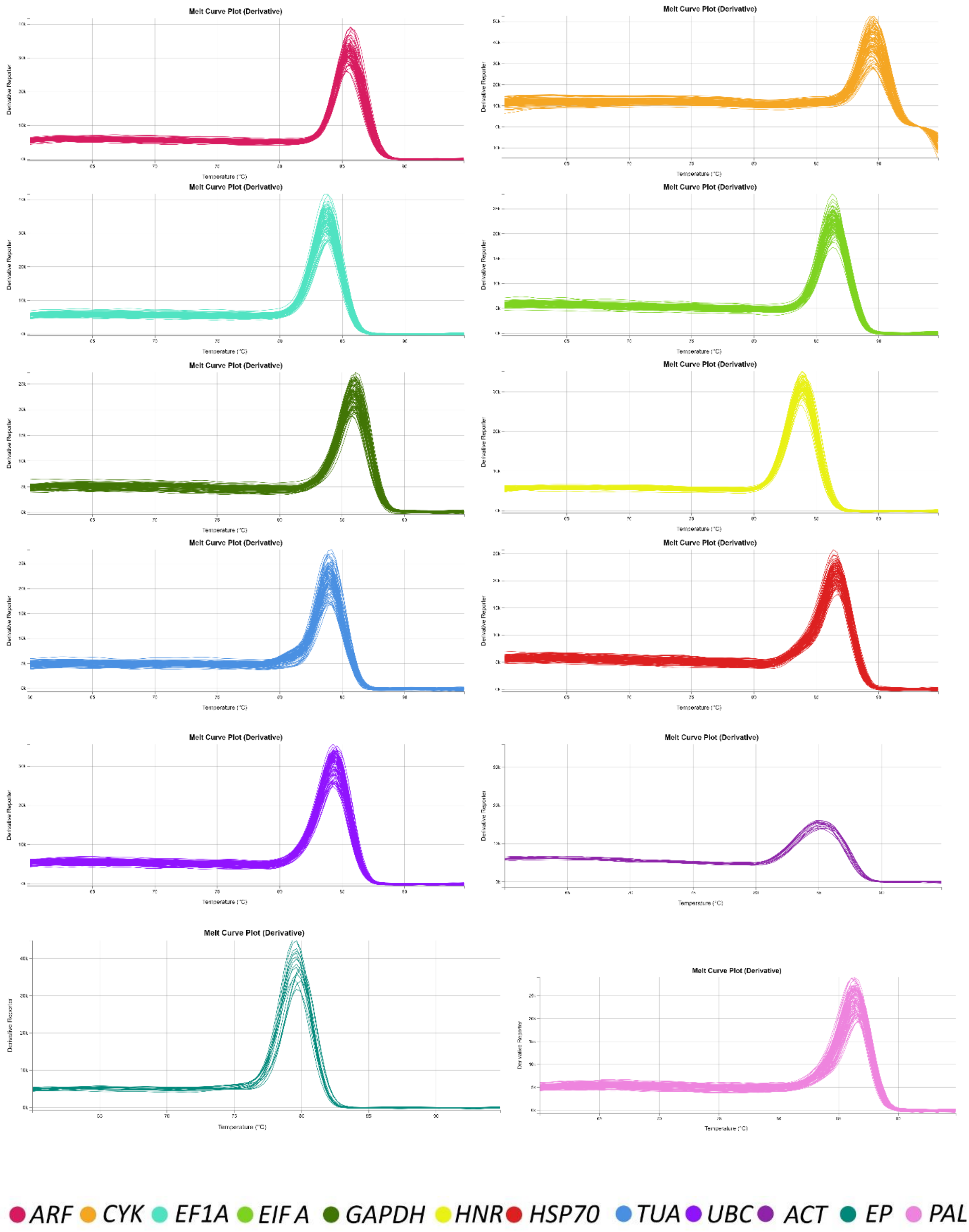

Fig. S1. Melt curves for RT-qPCR amplification of tested reference genes. *ARF* (ADP-ribosylation factor), *CYP* cyclophilin), *EF1A* (elongation factor 1-alpha), *EIF4A* (eukaryotic initiation factor 4A-3), *GAPDH* (glyceraldehyde-3-phosphate dehydrogenase), *HNR* (heterogeneous nuclear ribonucleoprotein 27C), *HSP70* (heat shock protein), *TUA* (alpha tubulin), *UBC* (ubiquitin conjugating enzyme (E2)).

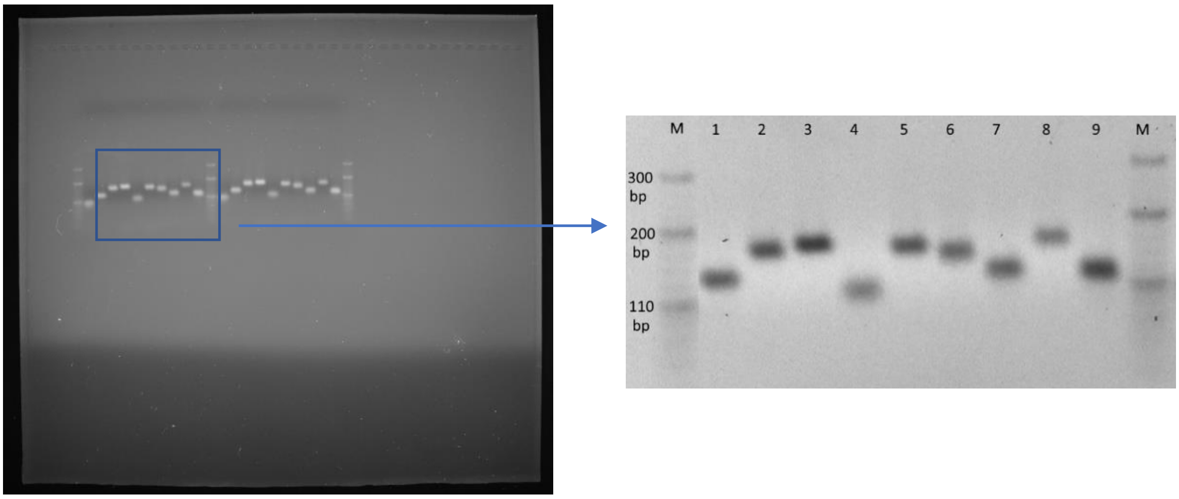

Fig. S2. cDNA amplification of nine candidate reference genes. M- 20bp Gene Ruler DNA ladder (Thermo Fisher Scientific Inc., USA). *(*1- *ARF* (ADP-ribosylation factor), 2- *CYP* cyclophilin), 3- *EF1A* (elongation factor 1-alpha), 4- *EIF4A* (eukaryotic initiation factor 4A-3), 5- *GAPDH* (glyceraldehyde-3-phosphate dehydrogenase), 6- *HNR* (heterogeneous nuclear ribonucleoprotein 27C), 7- *HSP70* (heat shock protein), 8- *TUA* (alpha tubulin), 9- *UBC* (ubiquitin conjugating enzyme (E2)))

Tab. S1. Primer sequences for candidate reference genes.

Gene symbol	Gene description	Primers/sequence source	Primer sequence 5'→3'	Amplicon size (bp)
ARF	ADP-ribosylation factor	XM_044532078.1 PREDICTED: *Triticum aestivum* ADP-ribosylation factor 1-like (LOC123111312), transcript variant X2, mRNA	F: GCCATGAATGCGGCTGAAA	113
			R: AGTCCCTCGTACAATCCCTC	
CYP	Cyclophilin	EU035525.*1* *Triticum aestivum* cyclophilin mRNA, complete cds	F: ATGGCCAACCCCAAGGTCT	145
			R: TCTTGCCCACGCCCTTCTC	
EF1A	Elongation factor 1-alpha	(Wrzesińska et al. 2016) KT153026.1 *Avena fatua* elongation factor 1-alpha (EF1) mRNA, partial cds	F: CAGGCAGATGATCTGCTGCT	158
			R: CCTCAAAGCCAGAGATTGGAA	
EIF4A	Eukaryotic initiation factor 4A-3,	(Yang et al. 2020) Locus_3892_Transcript_3/4_Confidence_0.667_Length_1160	F: TCTCGCAGGATACGGATGTCG	88
			R: TCCATCGCATTGGTCGCTCT	
GAPDH	glyceraldehyde-3-phosphate dehydrogenase	(Wrzesińska et al. 2016) KT153027.1 *Avena fatua* glyceraldehyde-3-phosphate dehydrogenase (GAPDH) mRNA, partial cds	F: CGTCAGGAACCCTGAAGAAA	146
			R: CTTTGCTAGGGGCTGAAATG	
HNR	Heterogeneous nuclear ribonucleoprotein 27C	(Yang et al. 2020) Locus_4951_Transcript_1/4_Confidence_0.667_Length_1455	F: ATTGGGTTTGTCACTTTCCGTAG	134
			R: CTTGGAGGGTGTCTCGCATCT	

HSP70	Heat shock protein	XM_003578850.3 PREDICTED: *Brachypodium distachyon* heat shock cognate 70 kDa protein 2 (LOC100843308), mRNA	F: ATCGACGCTGCCATTCAGTG<br>R: TCTTGGCGATGATGGGGTTG	109
TUA	Tubulin alpha	KT153029.1 *Avena fatua* alpha-tubulin (TUA) mRNA, partial cds	F: CGCCGCTCCCTTGATATTGA<br>R: TTGGGTAGGGCACCAGATTG	151
UBC	Ubiquitin conjugating enzyme (E2)	XM_044536383.1 PREDICTED*: Triticum aestivum* ubiquitin-conjugating enzyme E2 2 (LOC123115130), mRNA	F: CTGTGCGACCCGAATCCAAA<br>R: GCTCCACGATCTCACGAACT	103
ACT	Actin	(Wrzesińska et al. 2016)<br>KT153025.1 *Avena fatua* actin (ACT) mRNA, partial cds	F: CCGGTGATGGTGTGAGCC<br>R: CAGCGGTTGTTGTGAGGGA	150
EP	Expressed protein	(Yang et al. 2020)<br>Locus_827_Transcript_1/2_Confi-dence_1.000_Length_1359	F: CTTGGAGGGTGTCTCGCATCT<br>R: GCACAAGTGATGCCAGAATAGC	193
PAL	Phenylalanine ammonia lyase	(Tajti et al. 2021)<br>MT150275.1<br>*Triticum aestivum* phenylalanine ammonia-lyase (PAL) mRNA, complete cds	F: GCAACTTCCAGGGCACCC<br>R: CTCCGAGAACTGAGCGAACAT	95

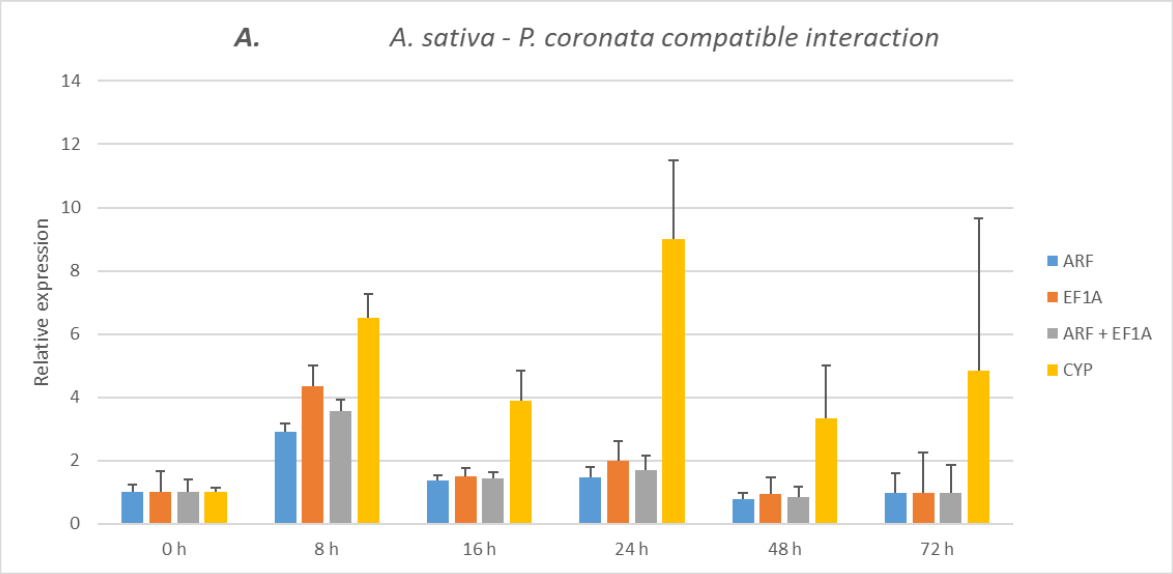

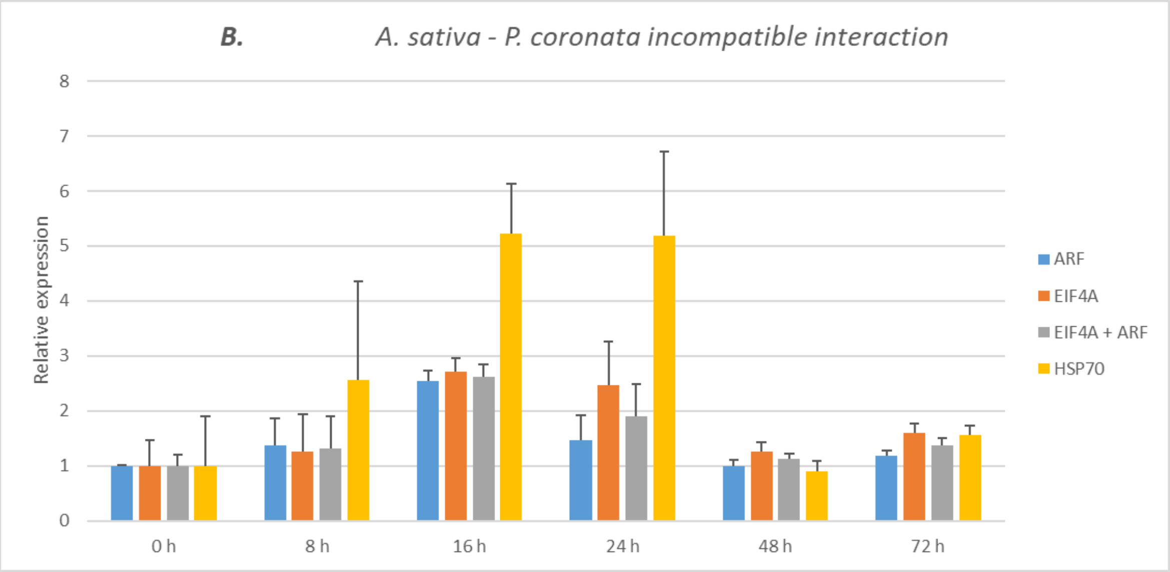

Fig. S3. Relative expression of the PAL gene following *P. coronata* f. sp.*avenae* inoclulation. Analysis was performed for *A. sativa* - *P. coronata* compatible (A) and incompatible (B) interaction against two best performing RGs (HNR and EF1A) separately or together as well as against worst performing RG (CYP). Data is shown as mean ± SD.
